# Supplementary material for: Single-Cell DNA Methylation Analysis of Chicken Lampbrush Chromosomes
Source: Int J Mol Sci. 2022 Oct 20;23(20):12601. doi: 10.3390/ijms232012601 (PMC9604247; doi:10.3390/ijms232012601)
Supplement: Supplementary file 1 [file ijms-23-12601-s001.zip › Supplementary_figures.pdf]

Single-cell DNA methylation analysis of chicken lampbrush chromosomes

Supplementary figures

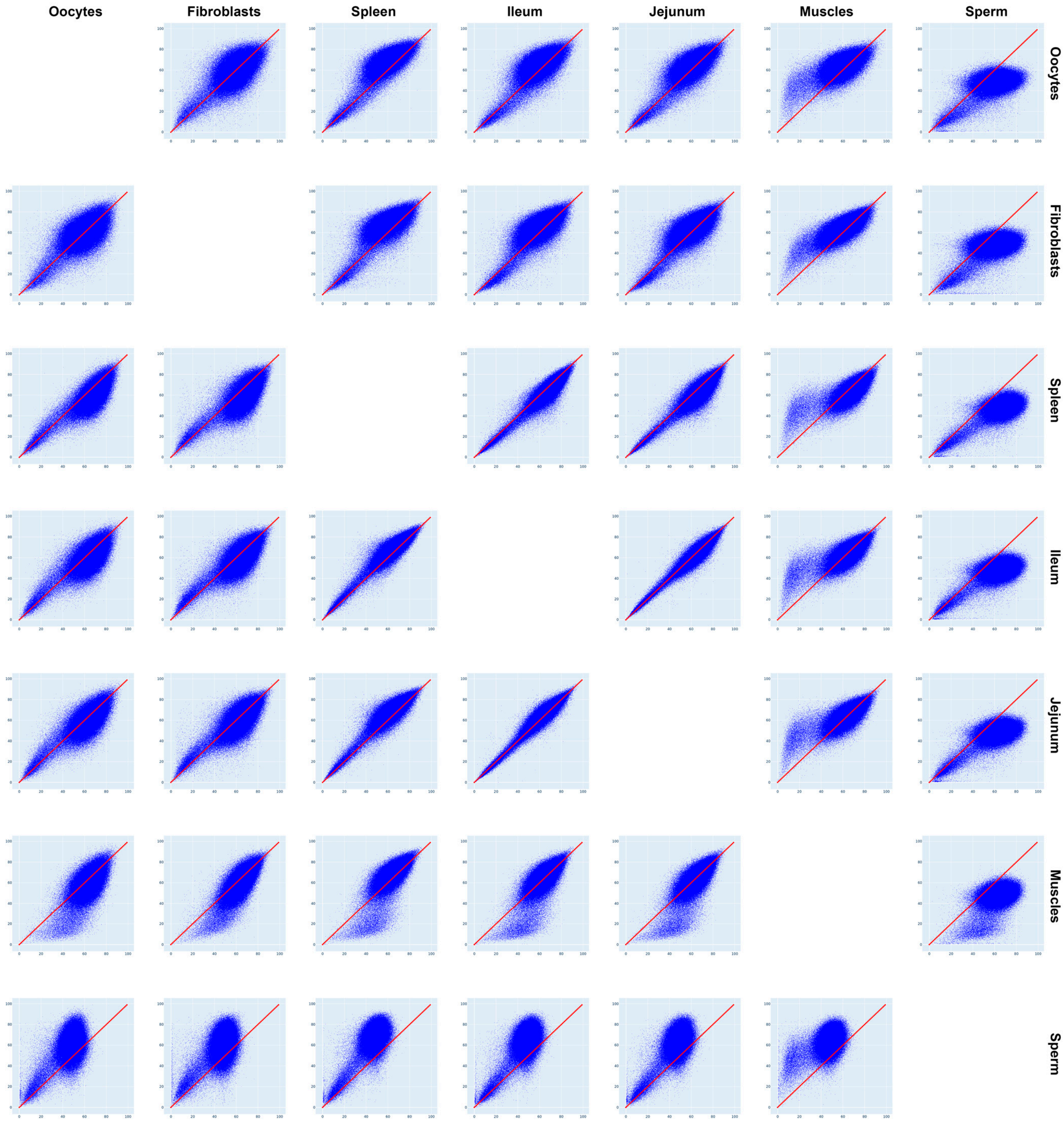

Figure S1. Pairwise methylation comparison of 5 kb genomic regions between different cell types. X and Y axes represent methylation levels in different cell types, each dot represents one 5 kb genomic bin.

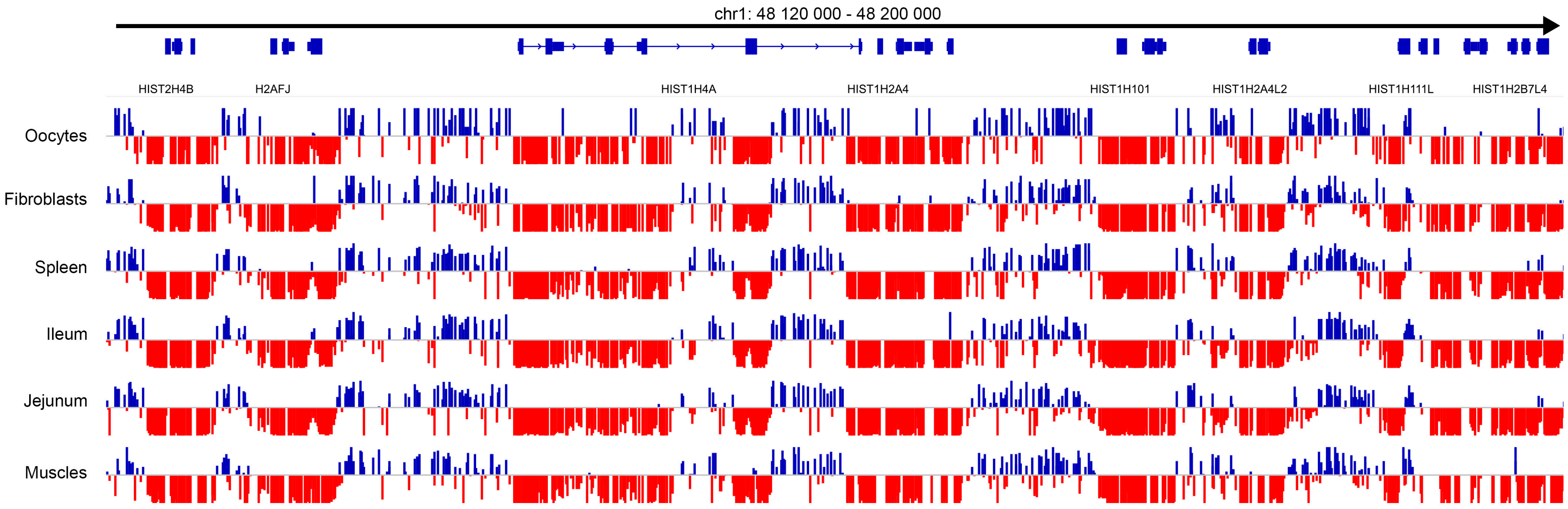

Figure S2. DNA methylation status of histone genes cluster in oocytes and other cell types.

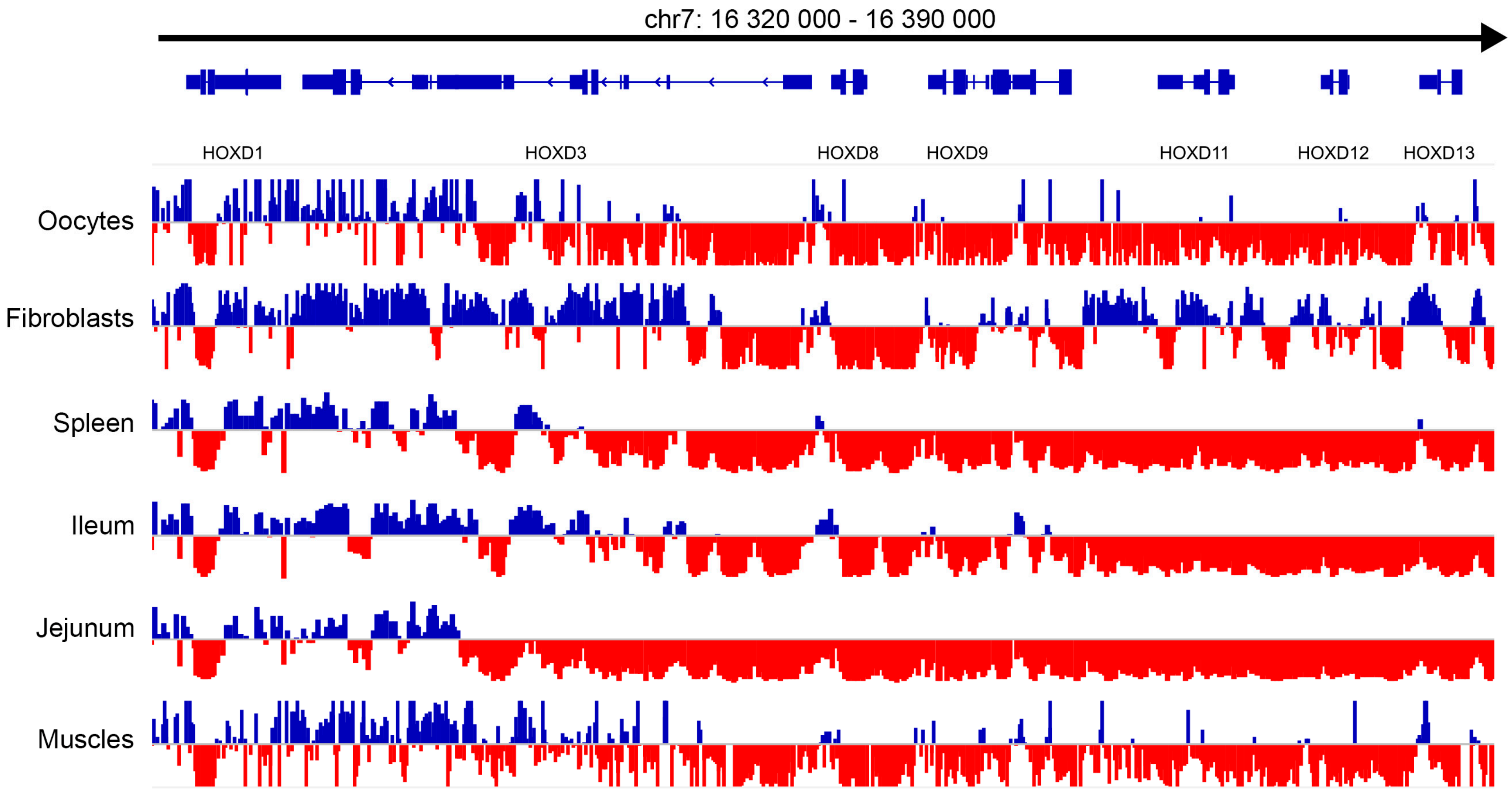

Figure S3. DNA methylation status of HoxD genes cluster in oocytes and other cell types.

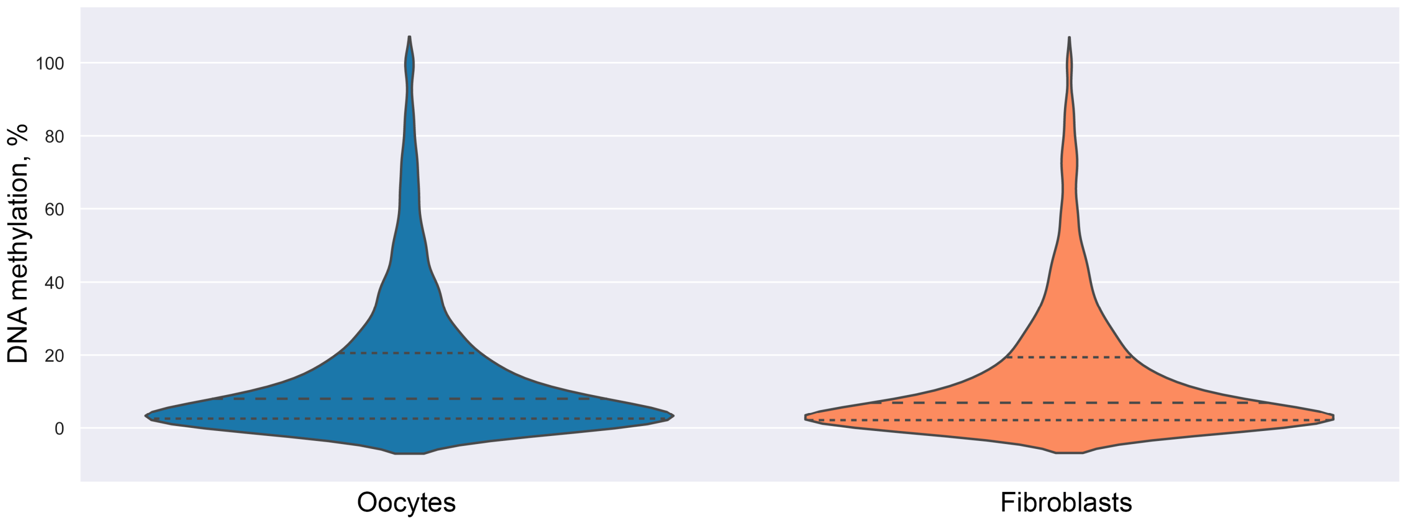

Figure S4. DNA methylation in CTCF binding sites – comparison between fibroblasts and diplotene oocytes. CTCF sites coordinates are taken from HD3 cell line ChIP-seq data (GSM1253766).

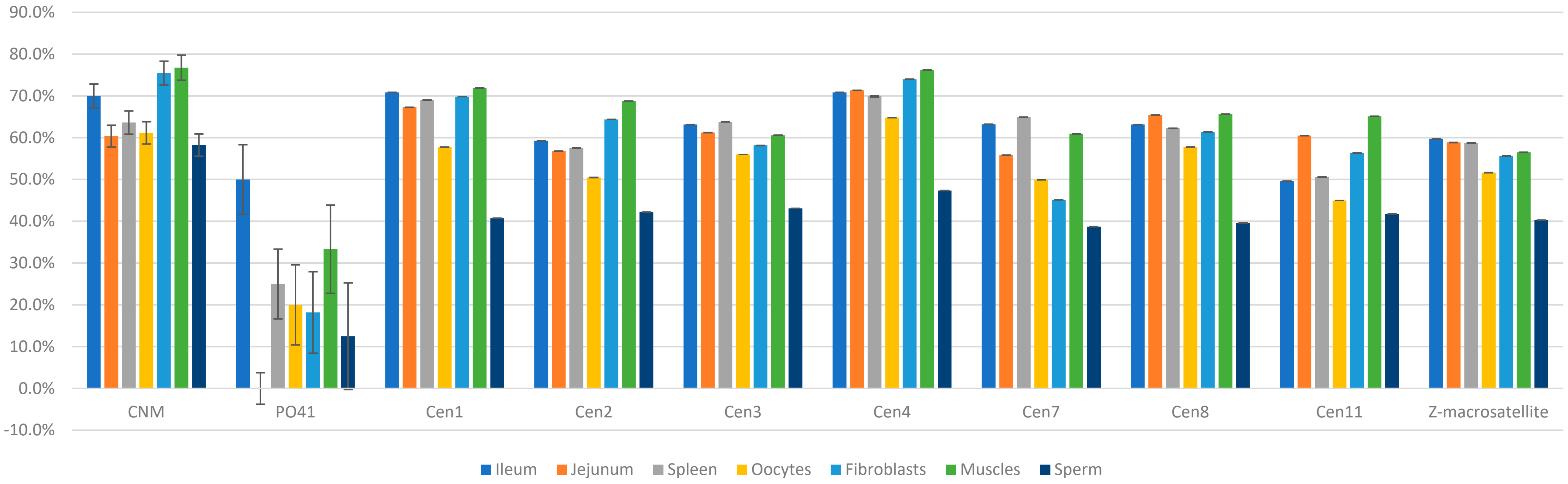

Figure S5. DNA methylation of avian-specific repeated elements across different chicken cell types.

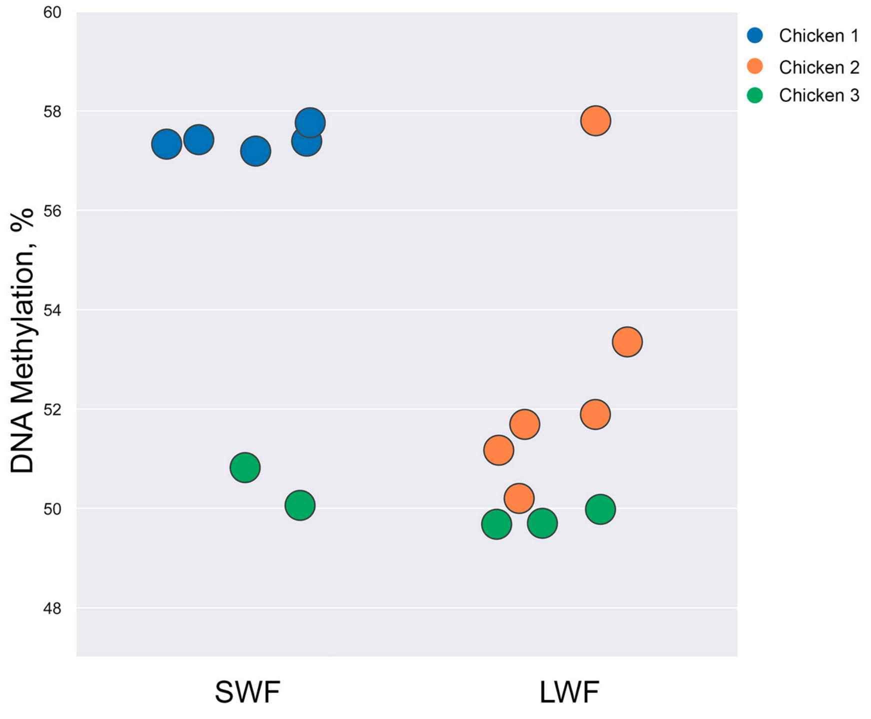

Figure S6. DNA methylation of chicken oocytes from different stages and animals. Data shows no significant difference in methylation between SWF and LWF stages (p=0.11).

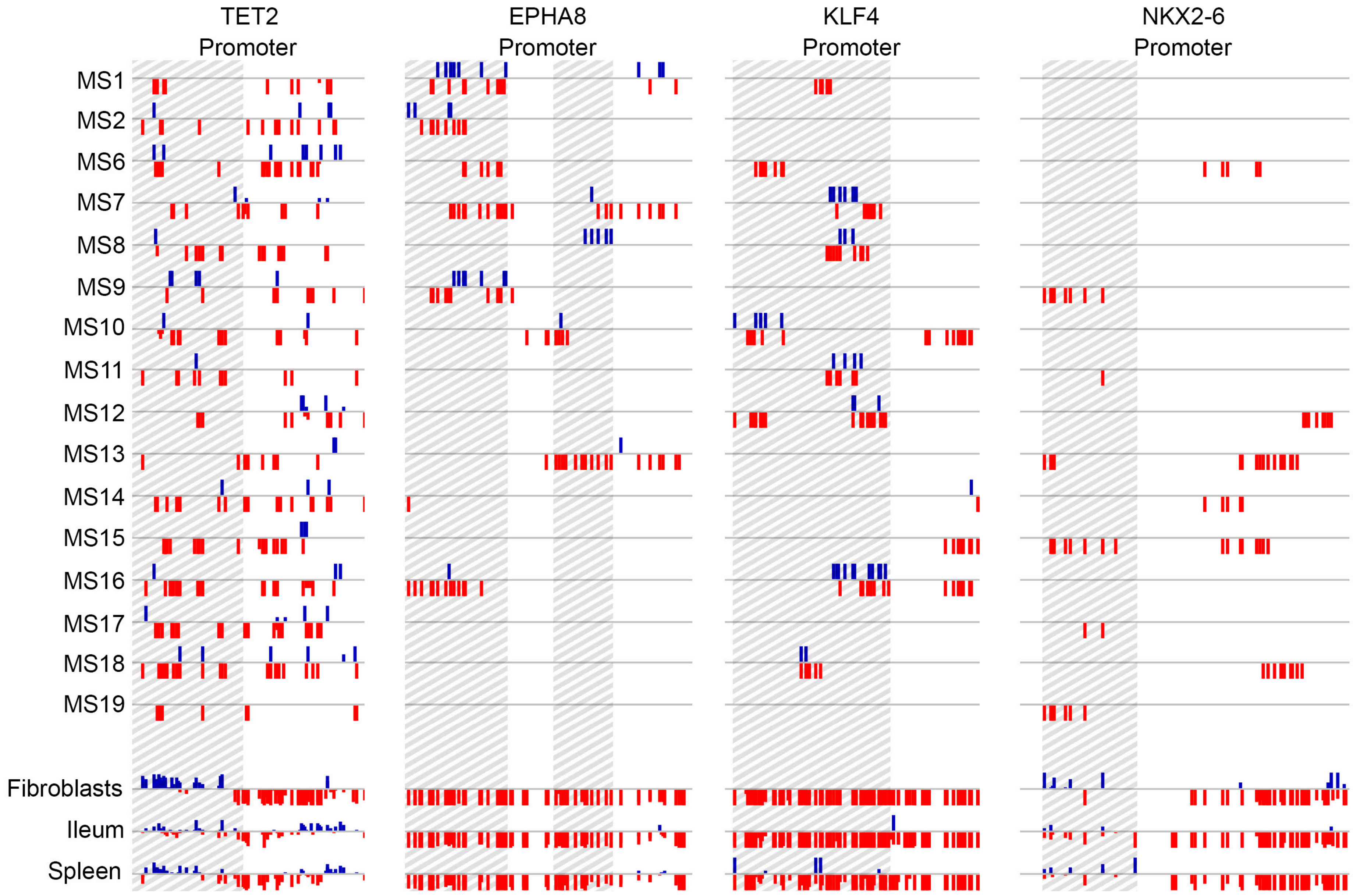

Figure S7. Examples of differentially methylated regions within promoters identified using HMM, single-cell samples are shown. Differentially methylated regions are highlighted.
