# Supplementary material for: Single-Cell DNA Methylation Analysis of Chicken Lampbrush Chromosomes
Source: Int J Mol Sci. 2022 Oct 20;23(20):12601. doi: 10.3390/ijms232012601 (PMC9604247; doi:10.3390/ijms232012601)
Supplement: Supplementary file 1 [file ijms-23-12601-s001.zip › Table S1.pdf]

Table S1. Sequencing summary for single-cell libraries.

Library	Oocyte stage	Mapped reads (single-end)	CpG covered	CpG methylation, %
MS1	SWF	4 233 467	3 745 339	57.33
MS2	SWF	4 294 346	4 065 140	57.19
MS6	SWF	3 761 381	3 846 393	57.39
MS7	SWF	3 917 920	3 765 203	57.76
MS8	SWF	3 525 490	3 524 664	57.42
MS9	LWF	2 294 949	2 248 514	51.69
MS10	LWF	3 537 999	3 022 951	50.20
MS11	LWF	4 000 781	2 183 219	57.80
MS12	LWF	2 552 973	2 212 127	53.35
MS13	LWF	4 277 809	2 889 211	51.89
MS14	LWF	2 748 091	2 579 421	51.17
MS15	SWF	4 297 209	2 512 089	50.82
MS16	SWF	3 571 361	3 310 282	50.06
MS17	LWF	4 179 621	3 853 446	49.68
MS18	LWF	2 969 772	2 611 373	49.98
MS19	LWF	1 502 800	1 505 668	49.70
