# Supplementary material for: Single-Cell DNA Methylation Analysis of Chicken Lampbrush Chromosomes
Source: Int J Mol Sci. 2022 Oct 20;23(20):12601. doi: 10.3390/ijms232012601 (PMC9604247; doi:10.3390/ijms232012601)
Supplement: Supplementary file 1 [file ijms-23-12601-s001.zip › Table S2.pdf]

Table S2. Statistics for HMM State 0 of DNA methylation for different chromosome types.

		Methylation								Length (in base pairs) for state N							
chr_type	state	count	mean	std	min	25%	50%	75%	max	count	mean	std	min	25%	50%	75%	max
W	0	54	3,914458	5,091115	0	0	0,940169	7,50974	17,2414	54	626,3704	597,042	22	189,25	417	821	2278
Z	0	2872	4,686931	4,481034	0	0	3,77358	7,8125	21,0526	2872	1007,481	1496,063	16	371,75	722	1288,25	45161
inter	0	7532	4,430319	4,264329	0	0,961538	3,15908	7,05516	33,3333	7532	663,314	732,9933	6	162	396	967	9501
macro	0	16695	4,168872	4,281295	0	0,770925	2,77778	6,54206	31,8182	16695	702,1552	770,2771	8	177	422	1008	15741
micro	0	13172	4,457402	4,157413	0	1,07527	3,26797	7,000973	24,2424	13172	638,0003	727,1959	6	158	371	894	12736
