# Supplementary material for: Single-Cell DNA Methylation Analysis of Chicken Lampbrush Chromosomes
Source: Int J Mol Sci. 2022 Oct 20;23(20):12601. doi: 10.3390/ijms232012601 (PMC9604247; doi:10.3390/ijms232012601)
Supplement: Supplementary file 1 [file ijms-23-12601-s001.zip › Table S3.pdf]

Table S3. Statistics for regions in which oocytes differ from other cell types (excluding sperm).

chr_type	state	count	mean	std	min	25%	50%	75%	max
W	0	16	379,9375	575,7534	22	70,75	166	275	1811
Z	0	1203	877,5278	823,1441	16	366,5	668	1166	10789
	1	67	432,8209	429,1282	11	92,5	268	555	1798
	2	2	87,5	78,48885	32	59,75	87,5	115,25	143
inter	0	1700	409,0188	438,0252	7	118,75	254	526,25	3598
	1	101	119,5644	114,6837	18	40	77	144	629
	2	3	37	27,73085	20	21	22	45,5	69
macro	0	3711	439,7141	477,7442	8	135	278	579,5	7975
	1	216	123,9769	155,0725	7	38,75	68	135,25	1038
	2	5	331,8	618,8067	23	25	66	108	1437
micro	0	2781	364,4412	411,6798	7	117	233	455	6135
	1	182	182,0769	243,9355	11	51	94,5	190,5	1581
	2	6	103,5	62,73675	30	60	95	139	199
